# Wellbeing, activity and housing satisfaction – comparing residents with psychiatric disabilities in supported housing and ordinary housing with support

**DOI:** 10.1186/s12888-017-1472-2

**Published:** 2017-08-30

**Authors:** Mona Eklund, Elisabeth Argentzell, Ulrika Bejerholm, Carina Tjörnstrand, David Brunt

**Affiliations:** 1 Department of Health Sciences/Mental Health, Activity and Participation (MAP), Lund University, Box 157, 221 00 Lund, SE Sweden; 2 Department of Health and Caring Sciences, Linneaus University, Växjö, Sweden

**Keywords:** Activity, Housing, Occupations, Psychiatric disabilities, Quality of life, Satisfaction

## Abstract

**Background:**

The home is imperative for the possibilities for meaningful everyday activities among people with psychiatric disabilities. Knowledge of whether such possibilities vary with type of housing and housing support might reveal areas for improved support. We aimed to compare people with psychiatric disabilities living in supported housing (SH) and ordinary housing with support (OHS) regarding perceived well-being, engaging and satisfying everyday activities, and perceived meaning of activity in one’s accommodation. The importance of these factors and socio-demographics for satisfaction with housing was also explored.

**Methods:**

This naturalistic cross-sectional study was conducted in municipalities and city districts (*n* = 21) in Sweden, and 155 SH residents and 111 OHS residents participated in an interview that included both self-reports and interviewer ratings. *T*-test and linear regression analysis were used.

**Results:**

The SH group expressed more psychological problems, but better health, quality of life and personal recovery compared to the OHS residents. The latter were rated as having less symptom severity, and higher levels of functioning and activity engagement. Both groups rated themselves as under-occupied in the domains of work, leisure, home management and self-care, but the SH residents less so regarding home management and self-care chores. Although the groups reported similar levels of activity, the SH group were more satisfied with everyday activities and rated their housing higher on possibilities for social interaction and personal development. The groups did not differ on access to activity in their homes. The participants generally reported sufficient access to activity, social interaction and personal development, but those who wanted more personal development in the OHS group outnumbered those who stated they received enough. Higher scores on satisfaction with daily occupations, access to organization and information, wanting more social interaction, and personal recovery predicted high satisfaction with housing in the regression model.

**Conclusion:**

The fact that health, quality of life and recovery were rated higher by the SH group, despite lower interviewer-ratings on symptoms and level of functioning, might partly be explained by better access to social interaction and personal development in the SH context. This should be acknowledged when planning the support to people who receive OHS.

## Background

The home is of fundamental importance for the possibilities for people with psychiatric disabilities to lead an everyday life characterized by meaningful activities, being as they spend a greater part of their time in their home setting [1]. The need to address opportunities for activity in the home context is evident from the research literature [1, 2], but has also emerged in countless contacts between our research group and staff working in housing services for people with psychiatric disabilities. Swedish time use research on persons with schizophrenia demonstrated that residents in supported housing had richer opportunities for social interaction and routines as opposed to those living on their own [2]. A cross-sectional study further revealed that higher levels of engagement in activities correlated with more satisfaction with the living situation [3]. To the best of our knowledge, no systematic description of activity opportunities in the homes of these people appears to exist.

Accommodation for people with psychiatric disabilities can vary from living in one’s own flat/house without support, often termed ordinary housing, to supported housing (SH), which links housing and support and is situated in supervised group homes or flats located in one building [4]. An intermediate form is ordinary housing with support (OHS), which is when a person living in an ordinary home receives support from a professional in order to manage that situation. Earlier research on housing for people with psychiatric disabilities has focused on the accommodation per se, by for example comparing institutional and community living [5] and addressing the individual’s preferences as to how they wish to live [6]. Their views on their living arrangements [7] and on aspects of the physical and psychosocial environments [4] have also been examined. However, research on activities in the home environment has received very little attention in Sweden and internationally. Research indicates that the residents in SH have severe psychiatric disabilities and the activity level in that context has been found to be low [8, 9]. Priebe et al. [10] reported on how support from the staff was adjusted to promote self-care activities in SH in Great Britain. A small-scale study in Sweden found that a method aimed at increased activity in the SH context resulted in good progress regarding both activity and health-related factors [11]. Considering the scarcity of research, however, more knowledge is needed regarding how those living in SH and those having OHS perceive their situation with regards to access to satisfying and engaging activities in their home environment. This type of descriptive and comparative research is important as a basis to generate knowledge that can be used to enrich the housing context and develop interventions aimed at enhanced opportunities for activity among residents. Previous research has shown that housing with optimal support can promote recovery from mental illness [12].

Activity is in this study defined as everyday life activities in a broad sense, including work-related activities, household work, self-care, and all kinds of recreational activities (physical, cultural and social). While work-related activities are mainly performed outside the home, taking care of one’s home, self-care and leisure activities often take place in the home [1, 2, 13–15]. There is convincing evidence of a strong connection between on the one hand meaningful, satisfying and engaging activities, and on the other health and well-being [16–19]. Meaningful activity is also an important aspect of recovery from mental illness [20]. This underlines the importance of addressing the residents’ perceptions of activity in the home context among people with psychiatric disabilities.

A growing body of research into housing for people with psychiatric disabilities in Sweden has revealed a number of significant differences between those who live in SH and those in OHS. In a study of satisfaction with housing, those in SH were more satisfied with their social life but less satisfied with the performance of support than those in OHS were [21]. Further indications of differences between the groups living in the two housing types are found in the results of two qualitative studies with Grounded Theory in the same project as above. The main concern of those in SH was “being deprived of self-determination”, focusing on the consequences of the organization and structure in the setting. On the other hand, the main concern of those in OHS was "the impossible mission in everyday life", focusing on their difficulties on trying to cope with a complex everyday existence [22, 23]. Further comparison of people in SH and those in OHS may provide additional indications regarding the type of support needed in the respective housing contexts.

## Study aim

Against the backdrop of research accounted for above, the current study aim was to compare people with psychiatric disabilities who live in two different housing contexts – SH and OHS – regarding perceived well-being, performance of engaging and satisfying everyday activities, perceived meaning of activity in one’s accommodation, and satisfaction with one’s current housing. As part of this aim, the importance of socio-demographic factors, perceived well-being, perceived everyday activities, and perceived meaning of activity in one’s accommodation for satisfaction with housing was explored.

## Methods

This was a cross-sectional, naturalistic study conducted in Sweden. The Regional Ethical Review Board in Lund approved the study, Reg. No. 2013/456.

### Study context

The two housing solutions featured in this study were SH and OHS. SH is a congregate residential facility where staff support can vary from office hours to 24 h per day, depending on the residents’ needs. The accommodation in SH can either consist of a fully-equipped flat in a housing block with similar flats or a bedroom and sitting room together with communal bathroom and eating facilities*.* OHS entails a flat or house the individual rents or owns in the ordinary housing market where he/she receives support from the municipal outreach housing services. The support is generally provided from once a week to once or twice per day depending on individual needs.

### Selection procedure

Four regions in Sweden were selected strategically according to variation in geographical location (southern, western, central and eastern regions). Within these regions, municipalities were selected according to size and socio-economic structure in order to attain variation among the participants. We approached major cities, suburban municipalities in their proximities, mid-sized and smaller towns, and rural municipalities. The final choice also took known characteristics regarding socioeconomic conditions into account (such as predominance of blue/white collar workers and proportion of immigrants). Two major cities that were approached had their administration decentralized to city districts. The managers of the SH units and the OHS teams in the selected municipalities/districts were contacted and invited to the study. A total of 27 municipalities/districts were approached and 21 agreed to participate. Reasons for declining were ongoing re-organization and recent participation in other projects. The participating municipalities/districts had several SH units and OHS teams, and maximum variation sampling [24] was applied to select the specific units. Variation was obtained on size (5–12 residents) and locations within the municipality/district. The managers had good insight into which SH units were well and less well-functioning (in terms leadership, education level among the staff and the unit’s psychosocial atmosphere) and variation was obtained regarding these criteria too. Almost all SH units provided 24-h support and more than two hours OHS support per week was rare.

Research assistants visited the SH units to inform about the study and the residents received both oral and written information. In the OHS context, the staff acted as gate-keepers and asked the residents about participation while also providing the written information. In both contexts, those who wanted to participate signed a written consent and provided their contact details. Because of the gate-keeper procedure it was not possible to keep exact track on the participation rate. Some of the gate-keepers asked only residents they thought would be willing to participate (led to higher participation rate) but some asked all potential participants. It was estimated that less than 50% of the eligible residents in SH agreed to participate, although the variation was great from one out of ten to nine out of ten. The participation rate was somewhat higher in the OHS group, approximately 50%.

### Procedure for data collection

One of the authors and ten research assistants with experience from working with people with psychiatric disabilities received specific training about using the instruments and performed the data collection. Nine of the data collectors were occupational therapists and the other two had an equivalent university education. The data collectors contacted the participants and scheduled a meeting. They were careful to create a trusting and calm atmosphere, and the participants were given the opportunity to choose where they felt most comfortable to meet. Most meetings took place in the participant’s home or in a common area of the housing facility. The instruments were administered in a certain order, and if needed the research assistant helped to explain the questions in other words and assisted in filling in the forms, while cautiously avoiding influencing any responses.

### Instruments

The target groups of this study, particularly those in SH, were expected to have limited endurance and concentration abilities and the selection of instruments was therefore kept as brief as possible. When available, one-item assessments were used, which has support in the literature on health ratings [25].

### Background questionnaire

A questionnaire was developed to address background data such as demographic factors, self-reported diagnosis, perceived health problems and any participation in activity-based day centers. Self-reported diagnosis was then classified by a psychiatrist according to the ICD-10 system [26]. The validity of the resulting ICD diagnoses has some support in previous research, in terms of logical differences on clinicians’ ratings of psychiatric symptoms when groups based on self-reported diagnoses were compared [27].

### Global assessment of functioning

To assess the overall functioning (severity of psychological, occupational and social functioning) of the participants, the Global Assessment of Functioning (GAF) scale [28] was used. The data collector rates the participant on a scale ranging between 1 and 100, where a higher score denotes better functioning. The GAF scale has proven to be a valid instrument and good inter-rater reliability has been demonstrated after a brief training of the data collector [29]. Those who performed the data collection for the present study had received training based on video cases and were calibrated against a highly experienced clinician until disagreement was reduced to <10%.

### Self-rated health

Three aspects of self-rated health were addressed, each with one item, using the first questions from the widely used MOS SF-36 [30]. The first concerned perceived current health, the second perceived current health compared to last year, and the third perceived current health compared to others of one’s age. A five-point scale was used, where a lower rating indicates better health. The first question has been proposed as a reliable one-item estimate of self-rated health [25]. We thus used that item but added two more items to include further facets of self-rated health. These items were analyzed separately and were not used as a scale.

#### Quality of life

The first item from the Manchester Short Assessment (MANSA) of quality of life [31, 32] was used to estimate general quality of life. This item has shown a high correlation with an index composed of domain-specific quality of life ratings, which is also part of the MANSA [31] but was not used for the current study. MANSA uses a seven-point rating scale from 1 (=worst possible satisfaction) to 7 (=best possible satisfaction).

### Self-mastery

Self-mastery has been defined as the experience of power over one’s life situation [33]. The Swedish version of the Pearlin Mastery Scale (Mastery-S), used for the present study, has proven to provide reliable data and represent a logical continuum of the construct [34]. It contains seven statements and a higher score implies a greater degree of self-mastery. The response format is a four-point scale that ranges from “Strongly disagree” (= 1) to “strongly agree” (= 4). Internal consistency based on the current sample was α = 0.76.

### Personal recovery

In order to measure personal recovery, the Process of Recovery Questionnaire (QPR) [35, 36] was used. The original QPR [35] is a 22-item questionnaire which comprises of two subscales; 1) intrapersonal tasks involved in recovery and 2) interpersonal factors that facilitate recovery. Seventeen of the items address the intrapersonal subscale and consist of different questions related to personal responsibilities and tasks related to recovery such as; “I can take charge of my life” and “I can actively engage with life”. The interpersonal subscale consists of five items and addresses thoughts on how recovery may be strengthened through interpersonal relationships, for example; “Meeting people who have had similar experiences makes me feel better”. The QPR items are each scored on a five-point scale (0 = disagree strongly, 1 = disagree, 2 = neither agree nor disagree, 3 = agree, 4 = agree strongly). A higher score indicates a higher level of personal recovery. The QPR has shown good construct validity in relation to well-being and quality of life and good test-retest reliability [35], but a brief version based only on the first subscale yielded even better psychometric properties [36]. A study developing a Swedish QPR version (QPR-Swe) arrived at the same conclusion [37]. For the current study, a shortened seven-item version of the QPR-Swe was used. Internal consistency reliability for that short-version based on the current sample was α = 0.82.

### Profiles of occupational engagement

To estimate the participants’ occupational engagement, the Profiles of Occupational Engagement in people with Severe mental illness (POES) was used. Occupation is here defined as all of the everyday activities people perform, including but not restricted to work. The instrument consists of two parts; a 24-h diary sheet, completed by the client, and an assessment scale, completed by a research assistant [38, 39]. The diary sheet is divided by one hour intervals and into four columns. Each column has a question at the top concerning the occupations performed, the social and geographical environments and about personal thoughts and feelings regarding the activity performed. The research assistant can ask prompting questions or provide assistance with writing. The assessment scale has nine items rated on a four-point scale (1 = low level of engagement and 4 = high level of engagement) and is based on the information in the diary sheet. Higher scores represent a higher level of occupational engagement. POES has proven to have satisfactory interrater agreement and construct validity [38, 40]. The research assistants received pre-training by the instrument developers. Cronbach’s alpha in the current study was α = 0.85. POES also includes a question asking whether the day registered in the diary was a typical one or not and 85% reported it being a typical day.

### Satisfaction with daily occupations and occupational balance

Satisfaction with daily occupations and occupational balance (SDO-OB) was used in order to measure satisfaction with everyday occupations, occupational balance and activity level. It covers four domains of everyday occupations: work, leisure, household chores and self-care. Satisfaction with the performance, or non-performance, within these different areas is reported by responding to 13 items. Each item has two parts, the first being yes/no questions asking whether or not the respondent presently performs the activity targeted in the item. These items produce an activity level score (yes = 1, no = 0), where a higher score represents a higher activity level. The respondent then rates satisfaction with the targeted activity, using a seven-step scale that ranges from worst possible satisfaction (=1) to best possible satisfaction (=7). High scores denote greater satisfaction. The SDO-OB also contains an occupational balance item after each domain. This item addresses the view of the respondent regarding occupational balance and the response alternatives are: far too little (−2), too little (−1), just enough (0), too much (1) and far too much (2). The items addressing occupational balance together form the respondent’s profile of occupational balance. The SDO-OB has been psychometrically tested and initial evidence of construct validity has been obtained [41]. Internal consistency is relevant only for the satisfaction scale, which yielded α = 0.84 based on the current sample.

### Activities in one’s accommodation

The Perceived Meaning of Activity in Housing (PMA-H) was developed by the research team with inspiration from the Estimating Perceived Meaning in Day Centers [42]. The PMA-H has 48 items, formulated as statements, all of which are preceded by the anchor “My housing contributes to…” [43]. Eleven items address the area of access to activity (such as “… that I can do activities that are good for me”), 12 target social interaction (such as “… I get a feeling of belonging in a group”), 13 concern possibilities for developing as a person (such as “… that I feel competent at something”) and 12 address organization and information (such as “… that I get information about rules and regulations here”). The response scale has five scale steps from 1 (=very little) to 5 (=very much), and a higher rating indicates greater activity meaning. Regarding the last area, organization and information, only six of the items are applicable for persons who have OHS. This study thus used only those six items. The areas form four subscales and internal consistency varied from 0.82–0.92 in the current study. A psychometric study has indicated good content validity, utility and construct validity of the PMA-H and absence of floor and ceiling effects [43]. As part of the PMA-H, the participants also rated whether or not they desired more of the aspects of activity, social interaction, personal development, and information in regard to their housing, using the alternatives “less” (= − 1), “enough as it is” (=0) and “more” (=1) for each area.

### Satisfaction with housing

Inspired by a client satisfaction scale [44], a housing satisfaction questionnaire was developed for this study. In both study contexts, the residents were encouraged to think about all aspects of their accommodation; including their physical home and the support and help they received to be able to manage there. The housing satisfaction questionnaire has eight items. These correspond to the eight items in the client satisfaction scale [44] and are rated on a four-point scale, where a higher value denotes greater satisfaction. Sample items are “Do you have the type of housing you want?” and “How satisfied are you with the housing support you get?” Internal consistency reliability based on the present sample was α = 0.90.

### Power calculation

A power calculation was based on the Satisfaction with Daily Occupations (SDO) assessment [45]. A previous study found a mean difference of 0.5 points on the SDO between groups of people with mental illness who had varying structure to their everyday life [46]. We based our calculation on the means and standard deviations from that study and took the influence of clustering effects into account. We expected a mean of 12.5 participants from each cluster (municipalities/districts) and an ICC of 0.05 [47]. This resulted in 65 participants in each group, but since the SDO had not been used in the context of residential support we wanted more than this and thus aimed to include 100 participants in each group. The data collection proceeded until that number was reached in the OHS group (actually 111), and at that time the number of SH participants amounted to 155. The study was thus somewhat over-powered.

### Data analysis

Differences between participants were estimated with the independent samples *t*-test for continuous data and the Chi-square test for categorical data. Relationships were assessed by Pearson correlations. A stepwise linear regression model was performed with satisfaction with the current type of housing as the dependent factor and socio-demographic, health-related and activity-related factors as independent factors. An association with the dependent factor at *p* < 0.05 was set as the limit for inclusion of independent variables.

In case of missing values on an instrument with several items, a simple form of imputation was made if a participant had answered 75% or more of the items. Each individual’s own mean value, based on the answered items, was inserted to replace the missing value. The imputation rates for the various instruments were for Mastery-S 1%, QPR 1%, POES 0%, SDO-OB activity level 7%, SDO-OB satisfaction score 7%, PMA-H activity 2%, PMA-H social interaction 7%, PMA-H development 5%, PMA-H organization 3%, and housing satisfaction 2% of the participants.

The *p*-value for statistically significant findings was set at <0.05. The software used was the IBM SPSS version 23 [48].

## Results

### Participants

Descriptive statistics on socio-demographics that characterize the study participants are presented in Table 1. The two groups were comparable on several factors. The mean age in both groups was in the span of 46–48 years, and a large majority had a friend and were born in Sweden. The group differences consisted of a greater proportion of men among the SH residents, and a greater proportion of residents who were married/cohabiting and were parents among the OHS residents. Moreover, the SH residents had a lower education level and had lived in their current accommodation for a shorter time.

**Table 1:** Socio-demographic characteristics of the two groups of residents

	Supported housing *N* = 155	Ordinary housing with support *N* = 111	*P*-value
Mean age (SD)	48 (12)	46 (11)	ns.
Proportion of women	43%	62%	0.001
Married/cohabiting	1.3%	10.3%	<0.001
Is a parent	26%	41%	0.007
Has a friend	89%	82%	ns.
Born in Sweden	85%	83%	ns.
Education^a^			<0.001
Non-completed 9-year school	6.5%	1.9%	
Completed 9-year school	46%	25%	
Completed high school	38%	49%	
Completed college/university	10%	25%	
Years in current accommodation (SD)	6.3 (5.5)	11 (8.3)	<0.001

^a^Adjusted residuals indicate that those in SH more often had completed 9-year school and less often a college/university degree compared to the OHS group

### Health and well-being in the two groups

Almost everyone in the two groups reported having a psychiatric diagnosis, and a vast majority took psychotropic medication. The diagnoses differed between the groups, with psychoses being more common and diagnoses categorized as anxiety/mood disorders and “other” disorders less common in the SH group (Table 2). A larger proportion of the SH group reported psychological problems, but they still rated their general health better than the participants in the OHS group. The same was found for the item targeting perceived health in relation to others of the same age group. There was no difference between the groups regarding perceptions of current health compared to a year ago. Nor was there any difference for self-mastery. The SH group assessed their quality of life and personal recovery higher than those in the OHS group.

**Table 2:** Health and well-being in the two groups

	Supported housing	Ordinary housing with support	*P*-value
Reports having a psychiatric diagnosis	98%	99%	ns.
Diagnostic category			<0.001
Psychosis	62%	32%	
Anxiety/mood disorders	13%	27%	
Other (usually Asperger syndrome, attention deficit disorder or unspecified mental disorder)	25%	41%	
Reports psychological problems	66%	53%	ns.
Takes psychotropic medicine	94%	88%	ns.
Reports physical problems	44%	57%	0.047
Self-rated health, general (SD)^a^	3.2 (1.1)	3.7 (1)	<0.001
Self-rated health, compared last year (SD)^a^	2.3 (1.1)	2.5 (1.2)	ns.
Self-rated health, compared to others (SD)^a^	2.8 (1.1)	3.4 (1.1)	<0.001
Quality of life (SD)	4.9 (1.6)	4 (1.7)	<0.001
Self-mastery (SD)	19.2 (4.3)	18.2 (4.6)	0.03
Personal recovery (SD)	27.4 (4.4)	25 (6)	<0.001
GAF symptoms (SD)	43.6 (13.9)	53.9 (8.9)	<0.001
GAF functioning (SD)	42.8 (11.1)	51 (10.8)	<0.001

^a^A lower value indicates better health

Table 2 also shows that the research assistants’ assessments of GAF symptoms and functioning differed between the groups with those from the OHS group receiving higher ratings in both respects.

### Activity-related factors

Most participants in both groups took a daily walk and about half of both groups attended a day center (Table 3). Participants from the OHS group who visited a day center spent more hours there than their counterparts in the SH group. Regarding perceived occupational balance, the SH group scored higher than those in OHS on the home management and self-care domains but not on the work and leisure domains. All values were negative, however, indicating the participants in both groups were under-occupied and had too little to do, but the OHS group was more severely under-occupied.

**Table 3:** Activity factors in the two groups

	Supported housing	Ordinary housing with support	*P*-value
Takes a daily walk	79%	84%	ns.
Attends a day center	47%	54%	ns.
Hours per week in the day center	8.6 (8)	12.5 (11.3)	0.032
Occupational balance, work (SD) (from SDO-OB)	−0.34 (0.82)	−0.38 (0.8)	ns.
Occupational balance, leisure (SD) (from SDO-OB)	−0.33 (0.76)	−0.45 (0.82)	ns.
Occupational balance, home management (SD) (from SDO-OB)	−0.17 (0.68)	−0.39 (0.89)	0.044
Occupational balance, self-care (SD) (from SDO-OB)	−0.17 (0.5)	−0.56 (0.75)	<0.001
Activity level (SD) (from SDO-OB)	6.7 (2)	7.1 (2.3)	ns.
Satisfaction with daily occupations (SD) (from SDO-OB)	71.3 (14.6)	64.2 (14.7)	<0.001
Occupational engagement (SD) (from POES)	22.6 (5.7)	26.2 (7)	<0.001
Access to activity (from PMA-H)	39.4 (8.7)	38.9 (8.9)	ns.
Social interaction (from PMA-H)	41.7 (9.8)	36.1 (12)	<0.001
Possibilities for personal development (from PMA-H)	47 (10.1)	43.6 (11)	0.012
Organization and information (from PMA-H)	20.4 (5.9)	18.8 (6)	ns.

The reported activity level was similar in both groups (Table 3), whereas satisfaction with daily occupations was rated higher in the SH group. On the other hand, the research assistant’s rating of occupational engagement was higher for the participants with OHS.

### Activities in one’s accommodation

Table 3 also reports the findings regarding characteristics of one’s accommodation with a focus on perceived meaning of activities. The groups differed on perceived possibilities for social interaction and personal development. The OHS group scored lower on both of these subscales. The standard deviation indicated significant within-group variation in the OHS group in terms of social interaction, where the group difference was also the greatest. The groups did not differ on access to activity or organization and information.

The participants also rated whether or not they desired more of the areas targeted in the PMA-H. Table 4 shows the distribution separately for the two groups regarding wanting less, receiving sufficient and wanting more in these respects. In general, the participants in the two groups considered they had sufficient of the targeted areas, but in the OHS group the proportion that wanted more personal development was larger than those who stated they received enough of this. The one statistically significant difference between the two housing groups concerned a larger proportion of the OHS group wishing they received more information in relation to their housing.

**Table 4:** Wants regarding activity, social interaction, personal development, and organization and information in the two groups, based on PMA-H

PMA-H area		Wants less	Just enough	Wants more	*P*-value
Access to activity	SH	0.7%	68%	31.3%	ns.
	OHS	1.9%	67%	31.1%	
Social interaction	SH	0.7%	63.7%	35.6%	ns.
	OHS	1.8%	59.6%	38.5%	
Personal development	SH	2.1%	58%	39.9%	ns.
	OHS	0%	48.6%	51.4%	
Organization and information	SH	4.3%	66.9%	28.8%	0.036
	OHS	0%	61.3%	38.7%	

### Satisfaction with type of housing

There was no statistically significant difference between the groups regarding satisfaction with their current type of housing (*p* = 0.172). The mean ratings (SD) were 24.4 (5.3) for the SH group and 25.4 (5.5) for those who had OHS. The variables that reached a statistically significant association with satisfaction with the type of housing and were entered in the regression model were personal recovery (*r* = 0.32; *p* < 0.001), satisfaction with daily occupations (*r* = 0.3; *p* < 0.001), self-mastery (*r* = 0.17; p = <0.011), access to organization and information (*r* = 0.23; *p* = 0.001), wanting more social interaction (*r* = 0.18; *p* = 0.008) and self-rated current health (*r* = −0.13, *p* = 0.047). All other variables presented in Tables 2, 3 and 4 were non-significant in relation to satisfaction with type of housing. Satisfaction with daily occupations, access to organization and information, wanting more social interaction and personal recovery became significant in the linear regression model. These variables explained 14.1% of the variation in housing satisfaction. Satisfaction with everyday occupations alone explained 6.4%, access to organization and information another 3.1%, wanting more social interaction 2.7%, and personal recovery 1.9%. The model summary is found in Table 5.

**Table 5:** Findings from the linear stepwise regression analysis (final step) explaining satisfaction with the type of housing

	R	R square change	F change	*P*-value for F change
Satisfaction with daily occupations	0.254	0.064	13.816	<0.001
Access to organization and information	0.308	0.031	6.803	0.01
Wanting more social interaction	0.350	0.027	6.165	0.014
Personal recovery	0.375	0.019	4.306	0.039

## Discussion

The aim of this study was to compare people residing in SH and OHS regarding perceived well-being, performance of engaging and satisfying everyday activities, perceived meaning of activity in one’s housing, and satisfaction with one’s current housing. The importance of socio-demographic factors, perceived well-being and perceived everyday activities for satisfaction with housing was also explored. The findings revealed several differences between people residing in SH and OHS. Some of these concerned characteristics of the residents, such as more men, fewer with families, fewer with a higher education and a lower level of functioning among people who lived in SH. In short, these differences indicate that the participants of this study received a relevant level of housing support. The two housing groups also differed in regard to health and well-being. The SH groups perceived more psychological problems than the OHS group, which was an expected finding, whereas it was the other way around concerning physical problems. Possibly, the fact that the SH group always had staff close at hand made them feel their physical problems were seen to. Previous research has shown that those who visited day centers for people psychiatric disabilities, many of whom had OHS, sought both psychiatric and primary health care quite seldom [27].

This appears to be one of few studies to compare people in SH and OHS regarding well-being, and considering the lower level of functioning in the SH group one could expect a lower level of well-being in that group. However, the findings of this study indicate the opposite, where the SH group rated higher levels of health, quality of life and recovery. Killaspy and colleagues [49] obtained similar results and found that quality of life was rated higher by residents who received a higher level of support. They reasoned that the higher level of autonomy that comes with less support also may bring increased risks to the residents’ personal safety, which may negatively affect their quality of life. But the findings of the present study may also be explained by the difference in diagnoses – it has been shown that people with schizophrenia or other psychosis (more prevalent in the SH group) rate their quality of life higher than those who have a mood disorder or anxiety disorder [50]. The fact that the SH group also scored higher on access to social interaction and personal development in the housing context may further explain the group difference on well-being. Social interaction has been found essential for self-rated health and well-being in both the general population [51–53] and among people with mental illness [54]. The low ratings in the OHS group on the well-being factors must be seen as a warning, indicating they may possibly receive too little social support and opportunities for development to maintain a satisfying level of quality of life.

There was no statistically significant difference between the groups on self-mastery, which on face value could be seen to deviate from the findings in two studies with Grounded Theory in Sweden that indicated differences between the two groups. The main concern of those in SH was “being deprived of self-determination”, while for those in OHS it was the “impossible mission in everyday life” [22, 23]. On the other hand, these main concerns could also be interpreted as constituting different aspects of the concept of self-mastery, which are expressed by one group as concerning self-determination, while for those in OHS the focus is on the struggle to cope with everyday life. More detailed studies focusing on aspects of self-mastery are thus warranted to clarify this.

In regard to activity factors, the result pattern indicated that the SH group were less active than the OHS group. This was indicated by less activity engagement and fewer hours spent in a day center. However, the SH residents were still more satisfied with their everyday activities and felt less under-occupied. This underscores that performance of activities and satisfaction with that performance are separate phenomena [55]. Despite the fact that the OHS group seemed better off regarding access to activity, they again seemed underprivileged as deemed by their satisfaction with that situation. Living in a congregate context can perhaps generate greater satisfaction per se. One can speculate about whether the SH residents were just satisfied with their activities or whether they were satisfied that they were doing something in a social context, but that remains to be investigated.

It is important to note that both groups were under-occupied and did not indicate they felt they had a balance among their everyday activities. There was a difference between the groups regarding home management and self-care, where the SH residents felt less under-occupied compared to their OHS counterparts. Possibly the support the SH group received was concentrated around these areas. Considering that both home management and self-care are activities one can perform in the home context, the findings of similar levels of under-occupations in all four activity areas for the OHS may seem somewhat surprising. They did not show better balance in these areas than in the work and leisure areas. It is possible, however, that the SH residents’ inner gauge for just-enough activity was lower than that of the OHS residents. In all, there seems to be a gap for both groups between their needs for activity and the available opportunities, which is something that needs to be considered when planning and organizing housing support to these groups.

Regarding perceived meaning of activity in one’s accommodation, as assessed by PMA-H, the SH group rated both access to social interaction and possibilities for personal development higher than the OHS group. Access to activity and to organization and information were rated similarly in both groups. Again, the OHS residents seem disadvantaged compared to SH residents. When asked whether they wanted less or more of the areas reflected through the PMA-H, a majority in both groups stated they received just enough, except for more than 50% in the OHS group wanting more personal development. It was also obvious that very few in both groups wanted fewer activities and that around 30% or more wanted increases in all PMA-H areas compared to the current situation. The truth that so many in the SH context were satisfied with their current amount of activity and also as a group rated their satisfaction with everyday activity higher than the OHS group, despite lower actual activity engagement, must be seen against their lower level of functioning and more severe psychiatric symptoms. This underscores the necessity of finding the right level of activity to match service users’ capacities [56, 57]. The SH residents seem to have received a better matched support, compared to the OHS group, regarding both well-being and activity aspects.

Finally, the groups did not differ significantly on satisfaction with housing situation. The factors that predicted satisfaction with housing situation were satisfaction with everyday activities, access to organization and information, wanting more social interaction, and level of personal recovery. Well-being factors such as quality of life and self-mastery were not related with housing satisfaction, which is in line with previous research [58]. A relevant question in relation to the current findings is how to design the housing services in such a way that they can enhance satisfying activities, organizational aspects, information, social interaction and the recovery process. A growing body of research suggests that both engagement in activity and social interaction promote recovery [59]. Existing knowledge thus indicates that activity engagement and a yearning for social interaction might also be important for housing satisfaction indirectly, through its enhancement of personal recovery. Support towards meaningful activity and stimulating social encounters in the housing context would thus be an important task for the staff, regardless of whether they provide SH or OHS support. Focusing further on social interaction, there was a group difference on *access* (greater in the SH group), but no group difference on *wanting more* of social interaction, but the rating of wanting thus emanated from levels of social interaction that differed clearly between the groups. Research on the impact of social support suggests that not only family and friend networks, but also social encounters that come naturally in the community, such as when shopping or going to the pharmacy, play a role for community integration and recovery. This was shown by Townley and colleagues [60], who used the term distal supports to denote these naturally occurring social relationships. Staff in SH services could stimulate both social support within the SH and distal supports in the community to optimize the care. Andersson [61] described that, in order to act as a supportive relationship in SH, the staff should create a social climate where they express interest in the individual, show care and concern, and also have respect for the integrity of the individual. Andersson further showed that the general social climate in SH had a strong influence on how support was received and if it was seen as supportive or unsupportive by the residents. The OHS group, who have few or no spontaneous contacts with staff that could catch them at the “right moment”, would need guidance to maximize the possibilities the distal supports entail. That could increase their opportunities for both activity engagement and social interaction.

### Methodological considerations

This was a cross-sectional study and cause-effect relationships could not be established. This is a general drawback using cross-sectional data, but findings may be used to generate hypotheses for future studies. The fact that self-reported diagnoses were used may be criticized for accurateness, but a previous study indicated a logical pattern of findings when groups based on self-reported diagnosis were compared on psychiatric symptoms rated by a professional [27]. Type and dosage of psychotropic medication was not registered, which is another limitation.

Moreover, the choice of instruments should be discussed. Single-item measures are sometimes criticized, but several researchers have shown that they perform very similarly and produce virtually identical answers compared to multiple-item versions measuring the same constructs [25, 62]. Furthermore, a less well-tested instrument was used to assess housing satisfaction. We wanted to address the combination of satisfaction with the dwelling and the support. We also preferred a brief scale since we wanted to include quite a few instruments and some participants, mainly those in the SH group, were easily exhaustible. When this study commenced we found no scale that fulfilled these criteria and chose to develop the here used questionnaire based on a widely used satisfaction scale [44]. It seems to have worked well in the current study, as indicated by the excellent internal consistency obtained and that only few imputations were needed. The imputation procedure may also be debated. When several responses are missing for each individual, multiple imputation based on the whole data set is recommended to predict the best value for imputation. This was not deemed necessary in the current study, considering that imputation was only made when at least 75% of the items were completed and the mean of these items was the imputed value.

Finally, the participation rate was hard to estimate depending on the use of a gate-keeper system. It is likely that lower-functioning participants are under-represented in both the SH and the OHS group. Although this may have influenced the findings in some unknown way, this study came as close as possible towards also including severely ill persons with a psychiatric disability, as indicated by the low GAF values with large standard deviations in both samples. Measures to accomplish this was to keep the data collection kit as brief as possible, split the data collection on two occasions, insert breaks when needed and assist in filling in responses if requested. Nevertheless, the fact that the non-participation rate remains unknown weakens the possibility to draw safe conclusions. We still propose that this study has internal validity. The external validity must be regarded as limited, however, although it should be possible to generalize the findings to similar housing contexts in countries where the housing support is organized in a similar way to that in Sweden.

## Conclusion

The results show that individuals in SH gave higher estimates regarding quality of life and personal recovery. Diagnosis and symptomatology may play a role here, since psychoses were more common in the SH group and mood and neurotic disorders, which were more common in the OHS group, have shown to be associated with worse quality of life. There seems to be a greater need for those living in OHS to access interventions that can promote increasing personal recovery and quality of life. However, both groups rated themselves as under-occupied and as having too little to do, which implies a need for activity-based interventions in both groups.

The results also show that, in order to promote satisfaction with housing, it is important for decision-makers with responsibility for SH and OHS to prioritize goals that include opportunities for satisfying activities, for taking part in organizational issues and receiving information, for access to social support, and for personal recovery. The housing staff need knowledge about how to provide increased access to these resources for the residents. Decision-makers need to consider the necessity for measures to increase these types of knowledge among staff. Satisfaction with housing could thus improve in both OHS and SH.

## Abbreviations

GAF: Global Assessment of Functioning
ICD: International Classification of Diseases
MANSA: Manchester Short Assessment of Quality of Life
MOS SF-36: Medical Outcomes Study, Short Form-36
OHS: Ordinary housing with support
PMA-H: Perceived Meaning of Activity in Housing
POES: Profiles of Occupational Engagement among people with Severe mental illness
QPR: Process of Recovery Questionnaire
SDO-OB: Satisfaction with daily occupations and occupational balance
SH: Supported housing

## References

[1] Bejerholm U, Eklund M (2004). Time-use and occupational performance among persons with schizophrenia. Occup Ther Ment Health.

[2] Bejerholm U, Eklund M (2006). Engagement in occupations among men and women with schizophrenia. Occup Ther Int.

[3] Bejerholm U (2010). Relationships between occupational engagement and status of and satisfaction with sociodemographic factors in a group of people with schizophrenia. Scand J Occup Ther.

[4] Brunt D (2002). Supported housing in the community for persons with severe mental illness. Psychosocial environment, needs, quality of life and social network.

[5] Leff J, Trieman N (2000). Long-stay patients discharged from psychiatric hospitals. Social and clinical outcomes after five years in the community. The TAPS project 46. Br J Psychiatry.

[6] Owen C, Rutherford V, Jones M, Wright C, Tennant C, Smallman A (1996). Housing accommodation preferences of people with psychiatric disabilities. Psychiatr Serv.

[7] Walker R, Seasons M (2002). Supported housing for people with serious mental illness: resident perspectives on housing. Can J Commun Ment Health.

[8] Forsberg KA, Bjorkman T, Sandman PO, Sandlund M (2010). Influence of a lifestyle intervention among persons with a psychiatric disability: a cluster randomised controlled trail on symptoms, quality of life and sense of coherence. J Clin Nurs.

[9] Bartels SJ, Mueser KT, Miles KM (1997). Functional impairments in elderly patients with schizophrenia and major affective illness in the community: social skills, living skills, and behavior problems. Behav Ther.

[10] Priebe S, Saidi M, Want A, Mangalore R, Knapp M (2009). Housing services for people with mental disorders in England: patient characteristics, care provision and costs. Soc Psychiatry Psychiatr Epidemiol.

[11] Lindström M, Hariz G-M, Bernspång B (2012). Dealing with real-life challenges: outcome of a home-based occupational therapy intervention for people with severe psychiatric disability. OTJR.

[12] Carpenter-Song E, Hipolito MM, Whitley R (2012). “right here is an oasis”: how “recovery communities” contribute to recovery for people with serious mental illnesses. Psychiatr Rehabil J.

[13] Eklund M, Leufstadius C, Bejerholm U (2009). Time use among people with psychiatric disabilities: implications for practice. Psychiatr Rehabil J.

[14] Leufstadius C, Eklund M (2008). Time use among individuals with persistent mental illness: identifying risk factors for imbalance in daily activities. Scand J Occup Ther.

[15] Leufstadius C, Erlandsson LK, Eklund M (2006). Time use and daily activities in people with persistent mental illness. Occup Ther Int.

[16] Bejerholm U, Eklund M (2007). Occupational engagement in persons with schizophrenia: relationships to self-related variables, psychopathology, and quality of life. Am J Occup Ther.

[17] Eklund M, Hansson L, Bejerholm U (2001). Relationships between satisfaction with occupational factors and health-related variables in schizophrenia outpatients. Soc Psychiatry Psychiatr Epidemiol.

[18] Aubin G, Hachey R, Mercier C (1999). Meaning of daily activities and subjective quality of life in people with severe mental illness. Scand J Occup Therapy.

[19] Goldberg B, Britnell ES, Goldberg J (2002). The relationship between engagement in meaningful activities and quality of life in persons disabled by mental illness. Occup Ther Ment Health.

[20] Haertl K, Minato M (2006). Daily occupations of persons with mental illness: themes from Japan and America. Occup Ther Ment Health.

[21] Brolin R, Rask M, Syren S, Baigi A, Brunt DA (2015). Satisfaction with housing and housing support for people with psychiatric disabilities. Issues Ment Health Nurs.

[22] Brolin R, Brunt D, Rask M, Syren S, Sandgren A (2016). Striving for meaning-life in supported housing for people with psychiatric disabilities. Int J Qual Stud Health Well-being.

[23] Brolin R, Brunt D, Rask M, Syren S, Sandgren A (2016). Mastering everyday life in ordinary housing for people with psychiatric disabilities. Grounded Theory Review.

[24] Patton MQ: Qualitative evaluation and research methods, 3 edn. Thousand Oaks [CA]: Sage; 2001.

[25] Bowling A (2005). Just one question: if one question works, why ask several?. J Epidemiol Community Health.

[26] WHO (1993). The ICD-10 classification of mental and behavioural disorders.

[27] Eklund M, Sandlund M (2012). The life situation of people with persistent mental illness visiting day centers: a comparative study. Community Ment Health J.

[28] Endicott J, Spitzer RL, Fleiss JL, Cohen J (1976). The global assessment scale. A procedure for measuring overall severity of psychiatric disturbance. Arch Gen Psychiatry.

[29] Startup M, Jackson MC, Bendix S (2002). The concurrent validity of the global assessment of functioning (GAF). Br J Clin Psychol.

[30] Ware JE, Sherbourne CD (1992). The MOS 36-item short-form health survey (SF-36). I. Conceptual framework and item selection. Med Care.

[31] Priebe S, Huxley P, Knight S, Evans S (1999). Application and results of the Manchester short assessment of quality of life (MANSA). Int J Soc Psychiatry.

[32] Björkman T, Svensson B (2005). Quality of life in people with severe mental illness. Reliability and validity of the Manchester short assessment of quality of life (MANSA). Nord J Psychiatry.

[33] Pearlin LI, Schooler C (1978). The structure of coping. J Health Soc Behav.

[34] Eklund M, Erlandsson LK, Hagell P (2012). Psychometric properties of a Swedish version of the Pearlin mastery scale in people with mental illness and healthy people. Nord J Psychiatry.

[35] Neil ST, Kilbride M, Pitt L, Nothard S, Welford M, Sellwood W, Morrison AP (2009). The questionnaire about the process of recovery (QPR): a measurement tool developed in collaboration with service users. Psychosis.

[36] Law H, Neil ST, Dunn G, Morrison AP (2014). Psychometric properties of the questionnaire about the process of recovery (QPR). Schizophr Res.

[37] Argentzell E, Hultqvist J, Neil ST, Eklund M: Measuring personal recovery – psychometric properties of the Swedish questionnaire about the process of recovery (QPR-Swe). Nord J Psychiatry*,* in press 2017. 10.1080/08039488.2017.1346144 28696806

[38] Bejerholm U, Eklund M (2006). Construct validity of a newly developed instrument: profile of occupational engagement in people with schizophrenia, POES. Nord J Psychiatry.

[39] Bejerholm U, Hansson L, Eklund M (2006). Profiles of occupational engagement among people with schizophrenia: instrument development, content validity, inter-rater reliability, and internal consistency. Br J Occup Ther.

[40] Bejerholm U, Lundgren-Nilsson A (2015). Rasch analysis of the profiles of occupational engagement in people with severe mental illness (POES) instrument. Health Qual Life Outc.

[41] Eklund M, Argentzell E (2016). Perception of occupational balance by people with mental illness: a new methodology. Scand J Occup Ther.

[42] Nilsson I, Argentzell E, Sandlund M, Leufstadius C, Eklund M (2011). Measuring perceived meaningfulness in day centres for persons with mental illness. Scand J Occup Ther.

[43] Eklund M, Brunt D. Measuring opportunities for engaging in meaningful home-based activities in housing services for people with psychiatric disabilities: development of the perceived meaning of activity in housing (PMA-H). Eval Health Prof. 2017;1–17. doi:10.1177/0163278717727333. 10.1177/0163278717727333 28835117

[44] Larsen DL, Attkisson CC, Hargreaves WA, Nguyen TD (1979). Assessment of client/patient satisfaction: development of a general scale. Eval Program Plann.

[45] Eklund M, Backstrom M, Eakman A (2014). Psychometric properties and factor structure of the 13-item satisfaction with daily occupations scale when used with people with mental health problems. Health Quality Life Outc.

[46] Eklund M, Hansson L, Ahlqvist C (2004). The importance of work as compared to other forms of daily occupations for wellbeing and functioning among persons with long-term mental illness. Community Ment Health J.

[47] Murray DM (1998). Design and analysis of group-randomized trials.

[48] IBM SPSS Statistics 23 core system user’s guide [ftp://public.dhe.ibm.com/software/analytics/spss/documentation/statistics/23.0/en/client/Manuals/IBM_SPSS_Statistics_Core_System_User_Guide.pdf].

[49] Killaspy H, Priebe S, Bremner S, McCrone P, Dowling S, Harrison I, Krotofil J, McPherson P, Sandhu S, Arbuthnott M (2016). Quality of life, autonomy, satisfaction, and costs associated with mental health supported accommodation services in England: a national survey. Lancet Psychiatry.

[50] Priebe S, Reininghaus U, McCabe R, Burns T, Eklund M, Hansson L, Junghan U, Kallert T, van Nieuwenhuizen C, Ruggeri M (2010). Factors influencing subjective quality of life in patients with schizophrenia and other mental disorders: a pooled analysis. Schizophrenia Res.

[51] Mukerjee S (2013). An empirical analysis of the association between social interaction and self-rated health. Am J Health Promot.

[52] Windsor TD, Rioseco P, Fiori KL, Curtis RG, Booth H (2016). Structural and functional social network attributes moderate the association of self-rated health with mental health in midlife and older adults. Int Psychogeriatr / IPA.

[53] Kawachi I, Berkman LF (2001). Social ties and mental health. J Urban Health.

[54] Eklund M, Hansson L (2007). Social network among people with persistent mental illness: associations with sociodemographic, clinical and health-related factors. Int J Soc Psychiatry.

[55] Carswell A, McColl MA, Baptiste S, Law M, Polatajko H, Pollock N (2004). The Canadian occupational performance measure: a research and clinical literature review. Can J Occup Ther.

[56] Townsend EA, Polatajko HJ (2013). Enabling occupation II: Advancing an occupational therapy vision for health, well-being & justice through occupation.

[57] Wilcock AA, Hocking C (2015). An occupational perspective of health 3edn.

[58] Tsai J, Mares AS, Rosenheck RA (2012). Does housing chronically homeless adults lead to social integration?. Psychiatr Serv.

[59] Doroud N, Fossey E, Fortune T (2015). Recovery as an occupational journey: a scoping review exploring the links between occupational engagement and recovery for people with enduring mental health issues. Aust Occup Ther J.

[60] Townley G, Miller H, Kloos B (2013). A little goes a long way: the impact of distal social support on community integration and recovery of individuals with psychiatric disabilities. Am J Community Psychol.

[61] Andersson G (2016). What makes supportive relationships supportive? The social climate in supported housing for people with psychiatric disabilities. Soc Work Ment Health.

[62] Cheung F, Lucas RE (2014). Assessing the validity of single-item life satisfaction measures: results from three large samples. Qual Life Res.

